# Supplementary material for: Pyrolysis Kinetics of Byrsonima crassifolia Stone as Agro-Industrial Waste through Isoconversional Models
Source: Molecules. 2023 Jan 5;28(2):544. doi: 10.3390/molecules28020544 (PMC9862415; doi:10.3390/molecules28020544)
Supplement: Supplementary file 1 [file molecules-28-00544-s001.zip › molecules-2098361-supplementary.pdf]

Supplementary Materials

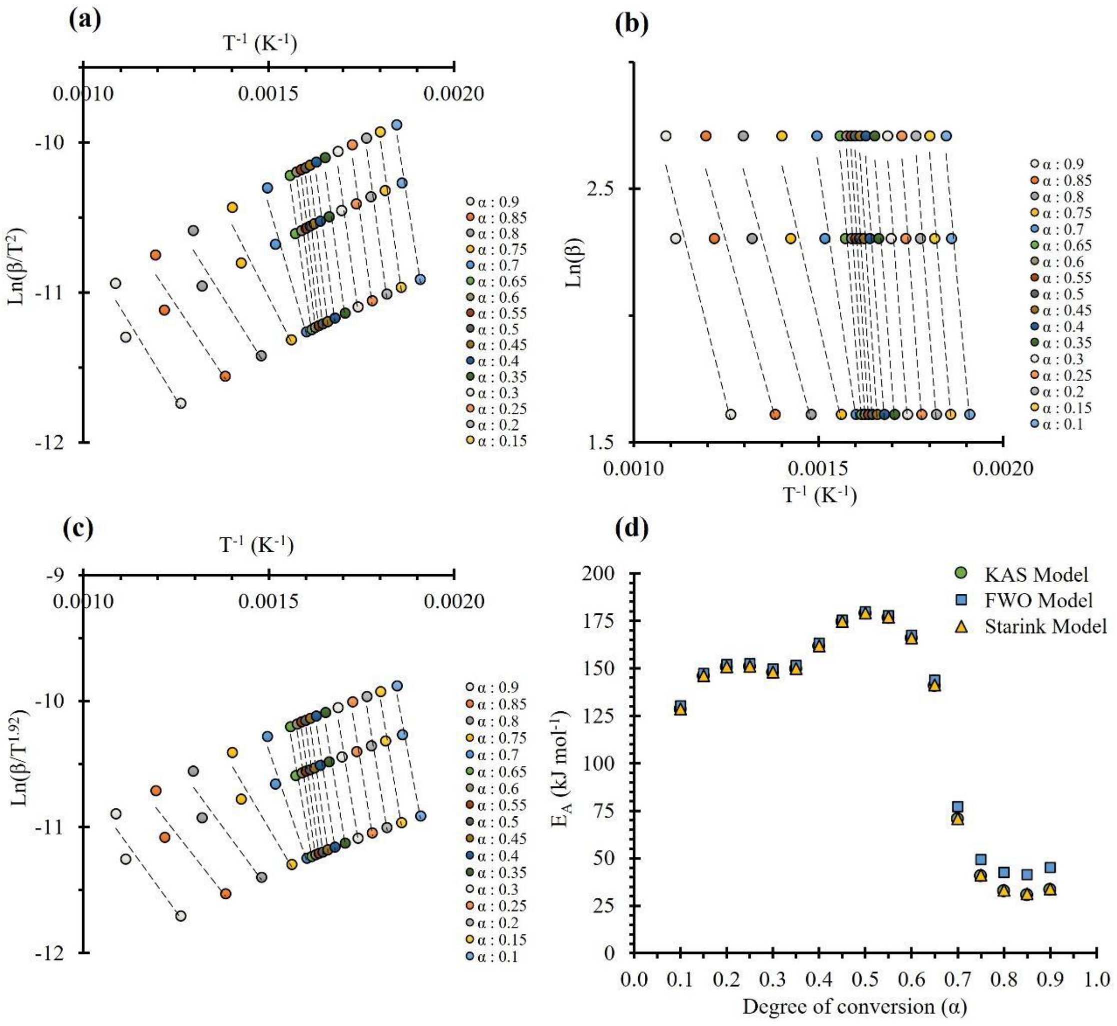

Figure S1. Linear plots of (a) KAS; (b) FWO model; (c) SK model: and (d) EA depending on the degree of conversion

Table S1.
Expressions for f(α) and g(α) functions of reaction models to describe thermal decomposition solid state reactions (Sangaré et al. and Mumbach et al.) [18, 19].

Reaction model		*f(α)*	*g(α)*
Reaction order models			
R1	Mampel (first order)	$1 - \alpha$	$-\ln(1 - \alpha)$
R2	Second order	$(1 - \alpha)^2$	$(1 - \alpha)^{-1} - 1$
R3	Third order	$(1 - \alpha)^3$	$0.5[(1 - \alpha)^{-2} - 1]$
R4	One and half order	$(1 - \alpha)^{3/2}$	$[(1 - \alpha)^{-1/2} - 1]$
Rn	n-order reaction	$(1 - \alpha)^n$	$[1-(1 - \alpha)^{1-n}]/(1-n)$
Diffusion models			
D1	One dimensional	$0.5\ \alpha^{-1}$	α^2
D2	Two dimensional (Valensi model)	$[-\ln(1 - \alpha)]^{-1}$	$(1 - \alpha)\ln(1 - \alpha) + \alpha$
D3	Diffusion control (Jander control)	$(1 - \alpha)^{2/3}\,[1 - (1 - \alpha)^{1/3}]^{-1}$	$[1 - (1 - \alpha)^{1/3}]^2$
D4	Diffusion control (Ginstling model)	$(3/2)\ [(1 - \alpha)^{-1/3} - 1]^{-1}$	$1 - (2/3)\alpha - (1 - \alpha)^{2/3}$
Power law nucleation models			
P1	Power law	$(2/3)\alpha^{-1/2}$	$\alpha^{3/2}$
P2	Power law	$2\alpha^{1/2}$	$\alpha^{1/2}$
P3	Power law	$3\alpha^{2/3}$	$\alpha^{1/3}$
P4	Power law	$4\alpha^{3/4}$	$\alpha^{1/4}$
Random nucleation and subsequent growth models			
A1	Avarami-Erofeev	$1.5(1 - \alpha)[-\ln(1 - \alpha)]^{1/3}$	$[-\ln(1 - \alpha)]^{2/3}$
A2	Avarami-Erofeev	$2(1 - \alpha)[-\ln(1 - \alpha)]^{1/2}$	$[-\ln(1 - \alpha)]^{1/2}$
A3	Avarami-Erofeev	$3(1 - \alpha)[-\ln(1 - \alpha)]^{2/3}$	$[-\ln(1 - \alpha)]^{1/3}$
A4	Avarami-Erofeev	$4(1 - \alpha)[-\ln(1 - \alpha)]^{3/4}$	$[-\ln(1 - \alpha)]^{1/4}$
A5	Random nucleation	$(1 - \alpha)^2$	$(1 - \alpha)^{-1}$
A6	Random nucleation	$0.5(1 - \alpha)^3$	$(1 - \alpha)^{-2}$
	Geometrical contraction models		
F2	Contracting cylinder	$2(1 - \alpha)^{1/3}$	$1 - (1 - \alpha)^{1/2}$
F3	Contracting sphere	$2(1 - \alpha)^{2/3}$	$1 - (1 - \alpha)^{1/3}$

Table S2.
Apparent activation energy (E_A) of pyrolysis of BCS determined by isoconversional methods.

	KAS		FWO		SK	
α	E_A (kJ/mol)	R^2	E_A (kJ/mol)	R^2	E_A (kJ/mol)	R^2
0.10	128.3	0.98	130.4	0.98	128.6	0.98
0.15	145.8	0.98	147.3	0.98	146.1	0.98
0.20	150.6	0.97	151.9	0.97	150.8	0.97
0.25	150.8	0.96	152.4	0.97	151.1	0.96
0.30	147.9	0.96	149.8	0.96	148.1	0.96
0.35	149.6	0.96	151.6	0.96	149.9	0.96
0.40	161.7	0.97	163.2	0.97	161.9	0.97
0.45	174.4	0.98	175.4	0.98	174.6	0.98
0.50	178.9	0.98	179.8	0.99	179.2	0.98
0.55	176.7	0.99	177.8	0.99	177.0	0.99
0.60	165.9	0.99	167.5	0.99	166.1	0.99
0.65	140.9	0.98	143.9	0.98	141.2	0.98
0.70	70.6	0.96	77.3	0.97	70.9	0.96
0.75	40.8	0.92	49.4	0.95	41.2	0.92
0.80	32.8	0.89	42.6	0.94	33.3	0.89
0.85	30.7	0.88	41.5	0.94	31.2	0.88
0.90	33.4	0.90	45.2	0.95	34.0	0.90
Average	122.3	0.95	126.3	0.97	122.7	0.96
